# Aneuploid Strawberry (2*n* = 10*x* − 3 = 67) Produces Variable Aneuploid Gametes and Fertile Hybrids with Improved Fruit Traits

**DOI:** 10.3390/plants15213402

**Published:** 2026-11-02

**Authors:** Juanjuan Chen, Tingting Xiong, Liangjie Li, Xiangxiang Dong, Shoufu Gong, Qingsong Zhu, Zhenfei Zhu, Min Zhai, Bailing Miao, Gangjun Luo

**Affiliations:** 1 College of Horticulture, Xinyang Agriculture and Forestry University, Xinyang 464000, China; 2019190001@xyafu.edu.cn (J.C.);; 2 School of Advanced Agriculture and Bioengineering, Yangtze Normal University, Fuling 408100, China

**Keywords:** strawberry, aneuploid, hybridization, chromosome number, aroma, fruit quality

## Abstract

For *Fragaria* (strawberry) species, individual chromosome identification is challenging due to the small size of chromosomes; consequently, the chromosome numbers of most cultivars remain unreported. In this study, we investigated the ploidy of 26 strawberry cultivars by counting root-tip chromosomes. Surprisingly, 25 cultivars were octoploid (2*n* = 8*x* = 56), whereas only ‘Tokun’ was aneuploid (2*n* = 10*x* − 3 = 67). To explore the breeding potential of this aneuploid, we performed reciprocal crosses between ‘Tokun’ and ‘Benihoppe’, a widely cultivated octoploid cultivar. Only the combination with ‘Benihoppe’ as female and ‘Tokun’ as male produced fertile progenies. The progenies had variable chromosome numbers of 61, 62 or 63, meaning that ‘Tokun’ produced aneuploid gametes with varying chromosome numbers ranging from 33 to 35. Some hybrids exhibited superior aroma, firmness, or soluble solids compared with their parents. Specifically, J2138 (2*n* = 9*x* − 2 = 61) was selected for its larger single-fruit weight, J2139 (2*n* = 9*x* − 2 = 61) and J2153 (2*n* = 9*x* − 2 = 61) for their white flesh, and J2151 (2*n* = 9*x* − 1 = 62) and J2156 (2*n* = 9*x* = 63) for their soluble solids content exceeding that of the higher parent, showing potential value for commercial production and breeding. Strawberry is usually propagated via stolons or through tissue culture; therefore, aneuploids obtained from 8*x* × aneuploid ‘Tokun’ crosses would be good sources for cultivar breeding.

## 1. Introduction

The cultivated strawberry (*Fragaria* × *ananassa* Duch.), an herbaceous perennial plant that belongs to genus *Fragaria* in the Rosaceae family, is one of the world’s most important berry crops, originating from occasional hybridization between *F. chiloensis* and *F. virginiana* in the 18th century [1,2,3]. The genus *Fragaria* exhibits a base chromosome number of seven (*x* = 7), with a naturally occurring polyploid series spanning from 2*x*, 4*x*, 5*x*, 6*x*, 8*x*, and 9*x* to 10*x* [4,5,6]. Additionally, 16*x* and 32*x* have been artificially induced using colchicine [1,7]. The extensive ploidy diversity within the genus *Fragaria* provides a solid genetic foundation for germplasm innovation.

To broaden genetic diversity and incorporate key agronomic traits, such as large fruit size, day neutrality, aroma and resistance to biotic and abiotic stresses, breeders have conducted extensive interspecific and interploidy hybridization in strawberry breeding, yielding pentaploids, hexaploids, heptaploids, nonaploids, and others [8,9,10,11,12,13,14], resulting in improved cold hardiness, disease resistance, and other agronomic traits in strawberry. Interspecific or interploidy hybridization is a key approach in strawberry breeding to break genetic barriers and achieve germplasm innovation; However, when the ploidy levels of the parental lines differ considerably, successful hybridization is difficult, and even when F_1_ progeny from distant crosses are obtained, they are generally highly sterile due to abnormal meiosis [15]. In some cases, although F_1_ offspring can be produced, they often produce small fruits with low commercial value [16,17,18,19,20]. Consequently, current strawberry breeding is largely confined to crosses among octoploid cultivars. However, this prolonged reliance on inter-cultivar hybridization has led to a progressively narrowing genetic base, with cultivated strawberry showing declining genetic diversity and reduced disease resistance, stress tolerance, and other agronomic traits.

Most strawberry genotypes are euploid, as their somatic cells possess complete chromosome sets that are exact integer multiples of the base number (*x* = 7). In contrast, aneuploids are characterized by somatic cells in which at least one genome exhibits an incomplete chromosome complement, resulting from the loss or gain of one or more chromosomes [21]. Henry et al. [22] confirmed that specific traits are closely associated with particular chromosome numbers. Due to the unbalanced changes in chromosome number, aneuploids exhibit a broad range of trait diversity [23,24,25,26], providing abundant selection materials for breeding [27]. However, there have been very few reports on aneuploidy in cultivated strawberry.

‘Tokun’, a newly released strawberry cultivar characterized by a peach-like fragrance, was developed through a multi-step breeding process. Initially, an interspecific hybrid was obtained from a cross between *F. nilgerrensis* (2*x*) and *F.* × *ananassa* (8*x*), followed by chromosome doubling with colchicine to generate decaploid (10*x*) lines. This cultivar was then bred by crossing two of these decaploid lines and selecting the superior progeny that retained the aromatic traits from *F. nilgerrensis* [15]. In the present study, we first conducted a chromosome survey of 26 strawberry cultivars to determine their ploidy levels and unexpectedly identified ‘Tokun’ as an aneuploid. To evaluate its breeding potential, we then crossed it with the octoploid cultivar ‘Benihoppe’ and assessed the ploidy levels and fruit quality traits of the progeny. This study aims to (1) determine the chromosome numbers of 26 strawberry cultivars, with a focus on the aneuploid ‘Tokun’ and its hybrid progeny, and (2) evaluate the feasibility of utilizing this aneuploid germplasm for trait introgression in strawberry.

## 2. Results

### 2.1. The Ploidy Levels of the 26 Strawberry Cultivars

**Figure 1:**
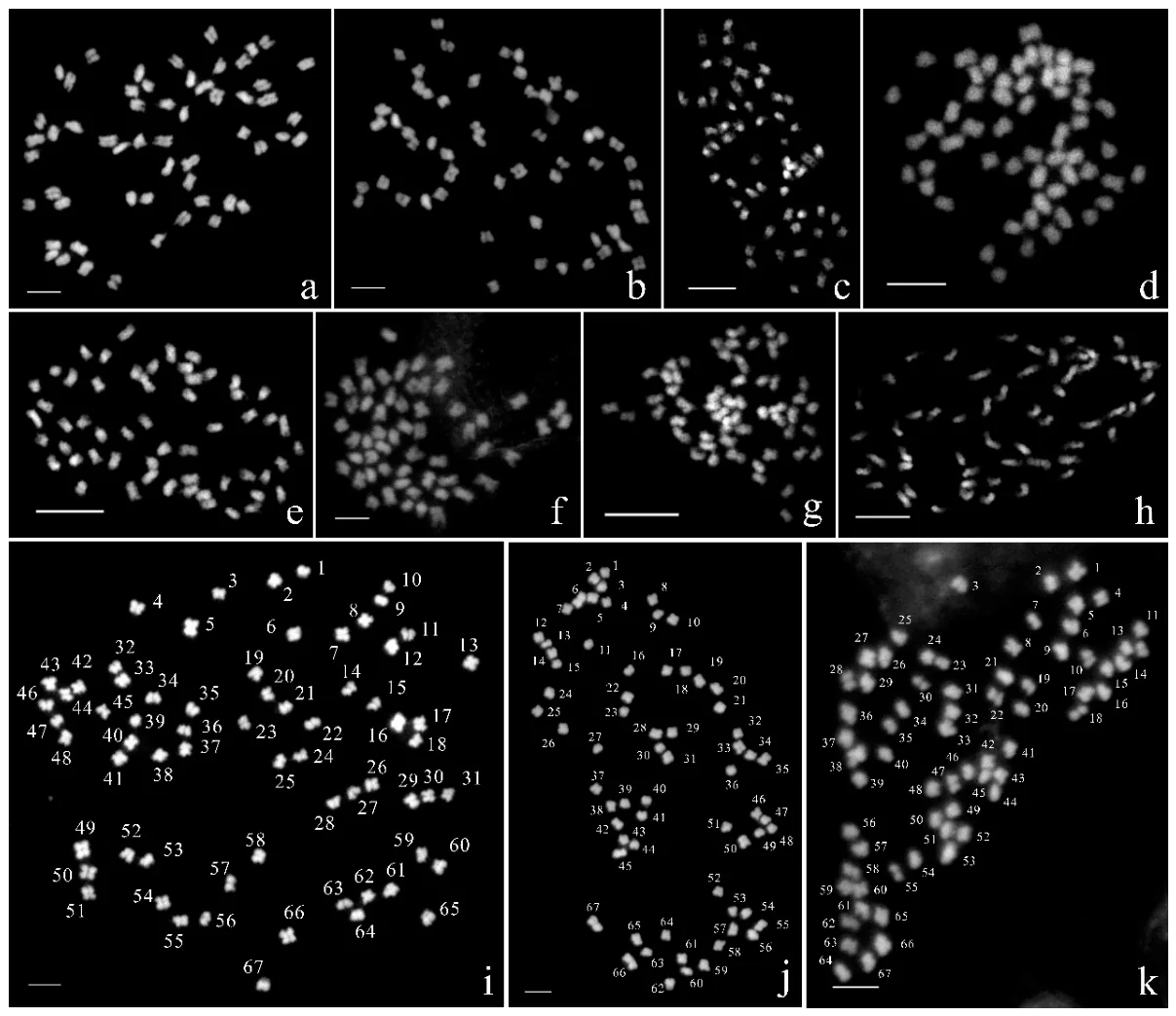
Chromosome numbers of nine cultivars. (**a**) ‘Benihoppe’; (**b**) ‘Yuehouji’; (**c**) ‘Ganlu’; (**d**) ‘Huangxiang’; (**e**) ‘Chandler’; (**f**) ‘Yutu’; (**g**) ‘Kaorino’; (**h**) ‘Guangdian’; (**i**–**k**) ‘Tokun’. Bars = 5 μm. The numbers in (**i**–**k**) indicate individual chromosomes counted in representative cells.

Chromosome counts were obtained for 26 strawberry cultivars. Among these, 25 were octoploid (2*n* = 8*x* = 56) (Figure 1a–h). Notably, in ‘Tokun’, all 20 well-spread metaphase cells examined from five plants showed 67 chromosomes (Figure 1i–k), which was reported for the first time (Table 1).

### 2.2. Fruit-Setting Rate and Seed Germination

Reciprocal crosses between ‘Benihoppe’ and ‘Tokun’ revealed substantial differences in fruit-setting rate and achene number per receptacle (Table 2). The fruit-setting rate (56.67%) and achene numbers per receptacle (25.71) of ‘Benihoppe’ × ‘Tokun’ were higher than those of ‘Tokun’ × ‘Benihoppe’ (26.67% and 19.63, respectively). A total of 116 seedlings were obtained from the cross of ‘Benihoppe’ × ‘Tokun’, while 15 weak seedlings were obtained when ‘Tokun’ was female parent, but they did not grow normally and eventually died before maturity.

### 2.3. Relative DNA Content and Chromosome Number of Parents and the Hybrids

**Figure 2:**
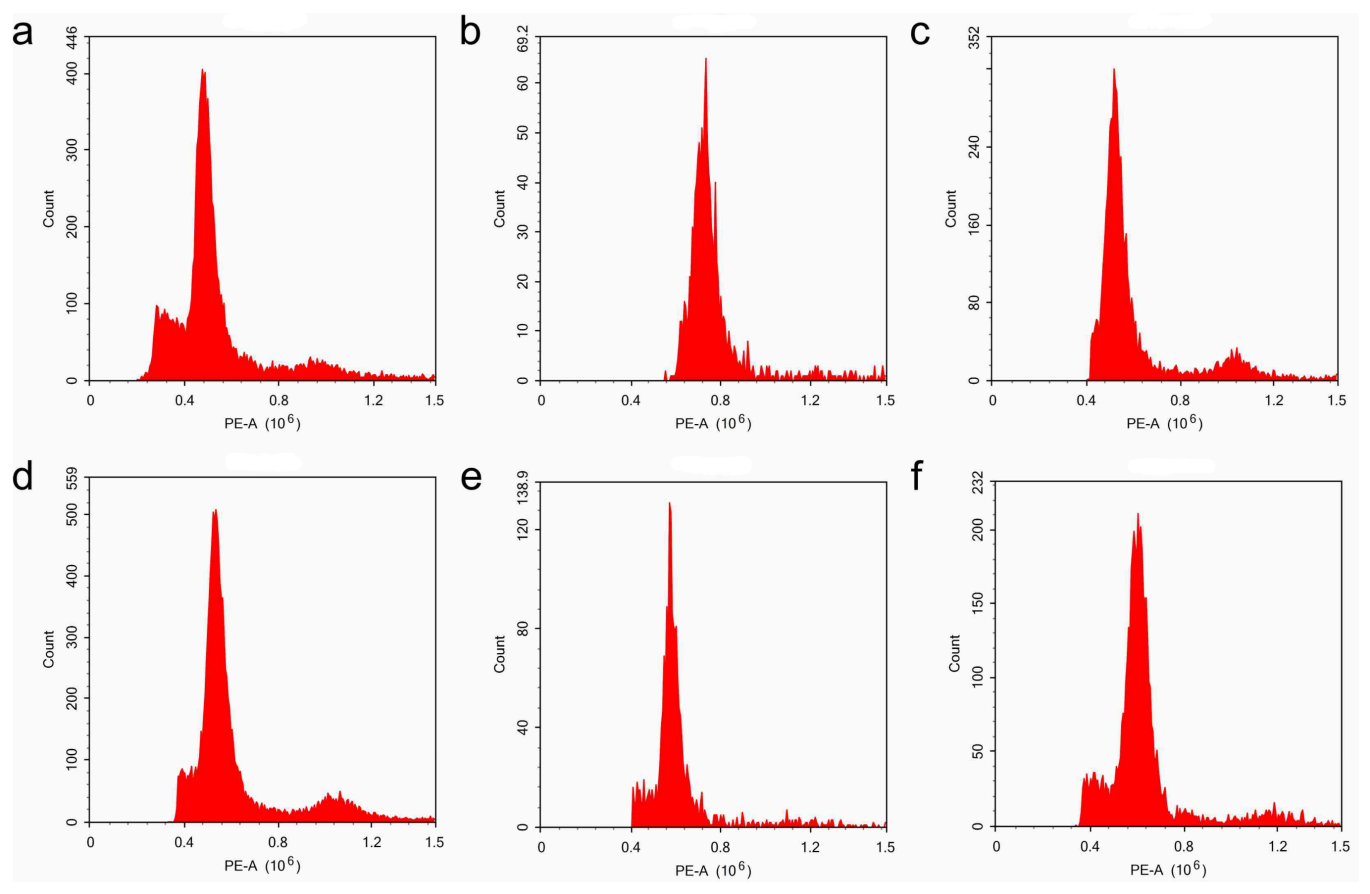
Relative fluorescence intensity of parents and selected hybrids analyzed by flow cytometry. (**a**) ‘Benihoppe’. (**b**) ‘Tokun’. (**c**) J2107. (**d**) J2101. (**e**) J2150. (**f**) J2156.

To accurately determine the genetic material of the parents and the hybrid progenies, both traditional chromosome counting and flow cytometry (FCM) were employed. ‘Benihoppe’ had a relative DNA content of 0.49 × 10^6^, while ‘Tokun’ showed a DNA content of 0.72 × 10^6^. The FCM histograms of these two parents were distinctly separated. The relative DNA contents of all hybrids ranged from 0.52 × 10^6^ to 0.63 × 10^6^, falling strictly between those of the two parents. This confirmed their true hybrid identity, demonstrating an intermediate inheritance pattern. Furthermore, the relative DNA contents of hybrids with 61, 62, and 63 chromosomes could not be distinguished (Figure 2).

**Figure 3:**
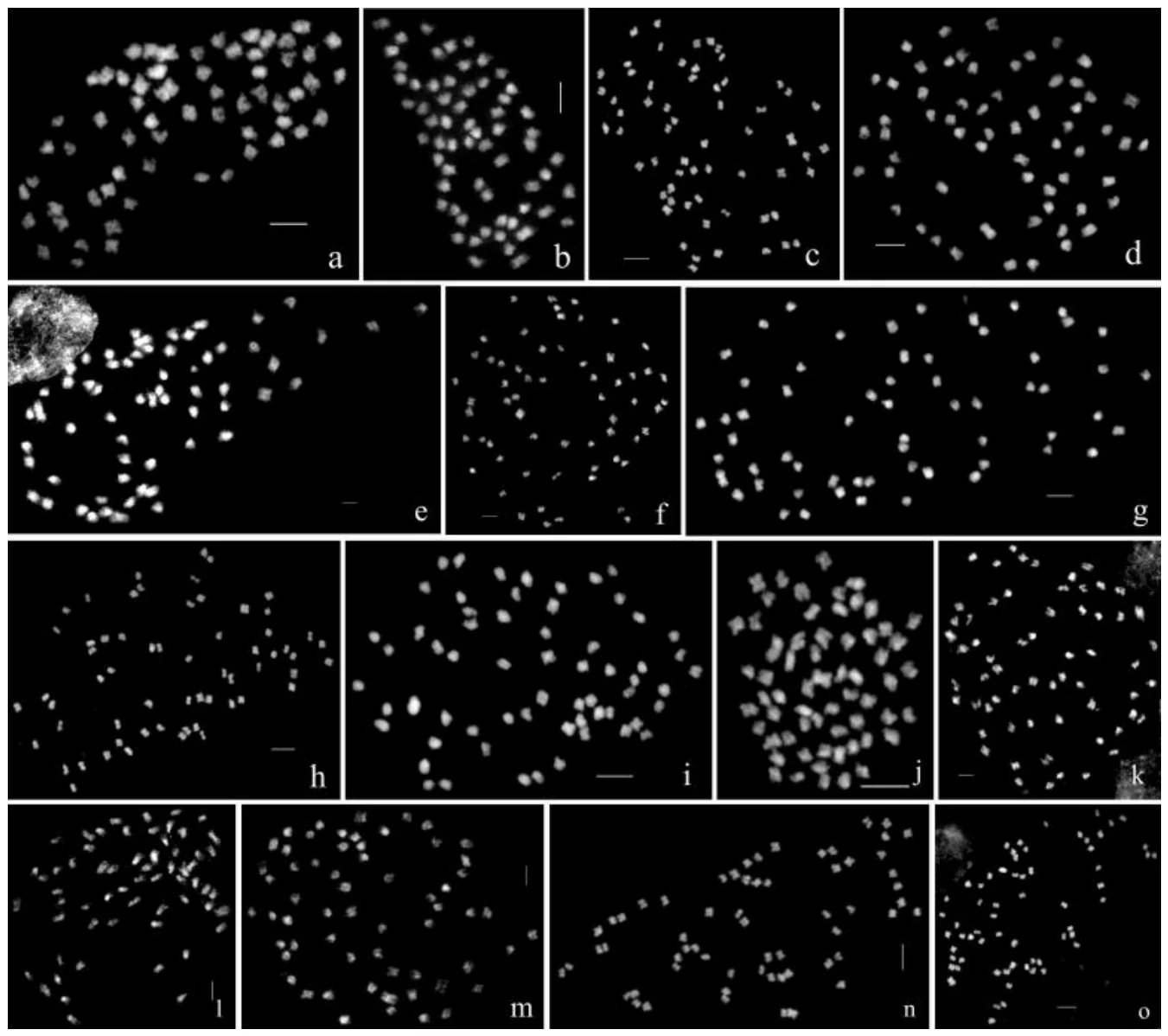
Chromosome numbers of root-tips in some hybrids from ‘Benihoppe’ × ‘Tokun’ and their parents. (**a**) J2101, 2*n* = 9*x *− 1 = 62. (**b**) J2105, 2*n* = 9*x* = 63. (**c**) J2107, 2*n* = 9*x *− 2 = 61. (**d**) J2125, 2*n* = 9*x *− 2 = 61. (**e**) J2127, 2*n* = 9*x *− 1 = 62. (**f**) J2132, 2*n* = 9*x *− 2 = 61. (**g**) J2138, 2*n* = 9*x *− 2 = 61. (**h**) J2139, 2*n* = 9*x *− 2 = 61. (**i**) J2140, 2*n* = 9*x *− 1 = 62. (**j**) J2150, 2*n* = 9*x* = 63. (**k**) J2151, 2*n* = 9*x *− 1 = 62. (**l**) J2153, 2*n* = 9*x *− 2 = 61. (**m**) J2154, 2*n* = 9*x *− 2 = 61. (**n**) J2155, 2*n* = 9*x *− 2 = 61. (**o**) J2156, 2*n* = 9*x* = 63. Bars = 5 μm.

**Figure 4:**
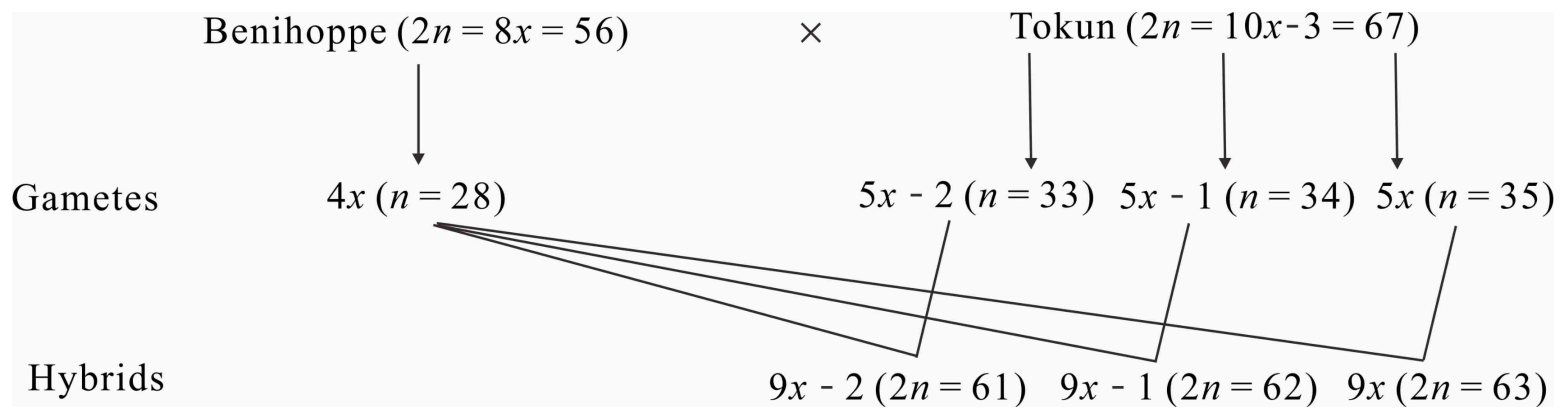
The putative origins of various ploidy hybrids from ‘Benihoppe’ × ‘Tokun’. The basic chromosome number of strawberry is *x* = 7. ‘Benihoppe’ (2*n* = 8*x* = 56) produces 4*x* (*n* = 28) gametes. ‘Tokun’ (2*n* = 10*x* − 3 = 67) produces variable gametes: 5*x *− 2 (*n* = 33), 5*x *− 1 (*n* = 34), and 5*x* (*n* = 35). Consequently, the resulting hybrids have chromosome numbers of 9*x* − 2 (2*n* = 61), 9*x* − 1 (2*n* = 62), and 9*x* (2*n* = 63).

The chromosome numbers of the observed seedlings are presented in Table 3. Among the 27 hybrids, the chromosome numbers of the seedlings were predominantly nonaploid minus one (40.74%) or two (48.15%), and only 11.11% were nonaploid, with an average of 61.63. No seedlings with nonaploid minus three, minus four, or minus five were observed (Figure 3, Table 3). Since octoploid parents undergoing regular meiosis rarely produce aneuploid gametes, the observed aneuploidy is attributable to irregular meiotic segregation in ‘Tokun’. The nonaploid (9*x*) and aneuploid hybrids were probably derived from the union of the 4*x* gamete of the female ‘Benihoppe’ and 5*x*, 5*x* − 1 and 5*x *− 2 gametes of the male ‘Tokun’ (Figure 4).

### 2.4. Pollen Germination, Flower and Stolon Traits, and Fruit Quality of the Hybrids

**Figure 5:**
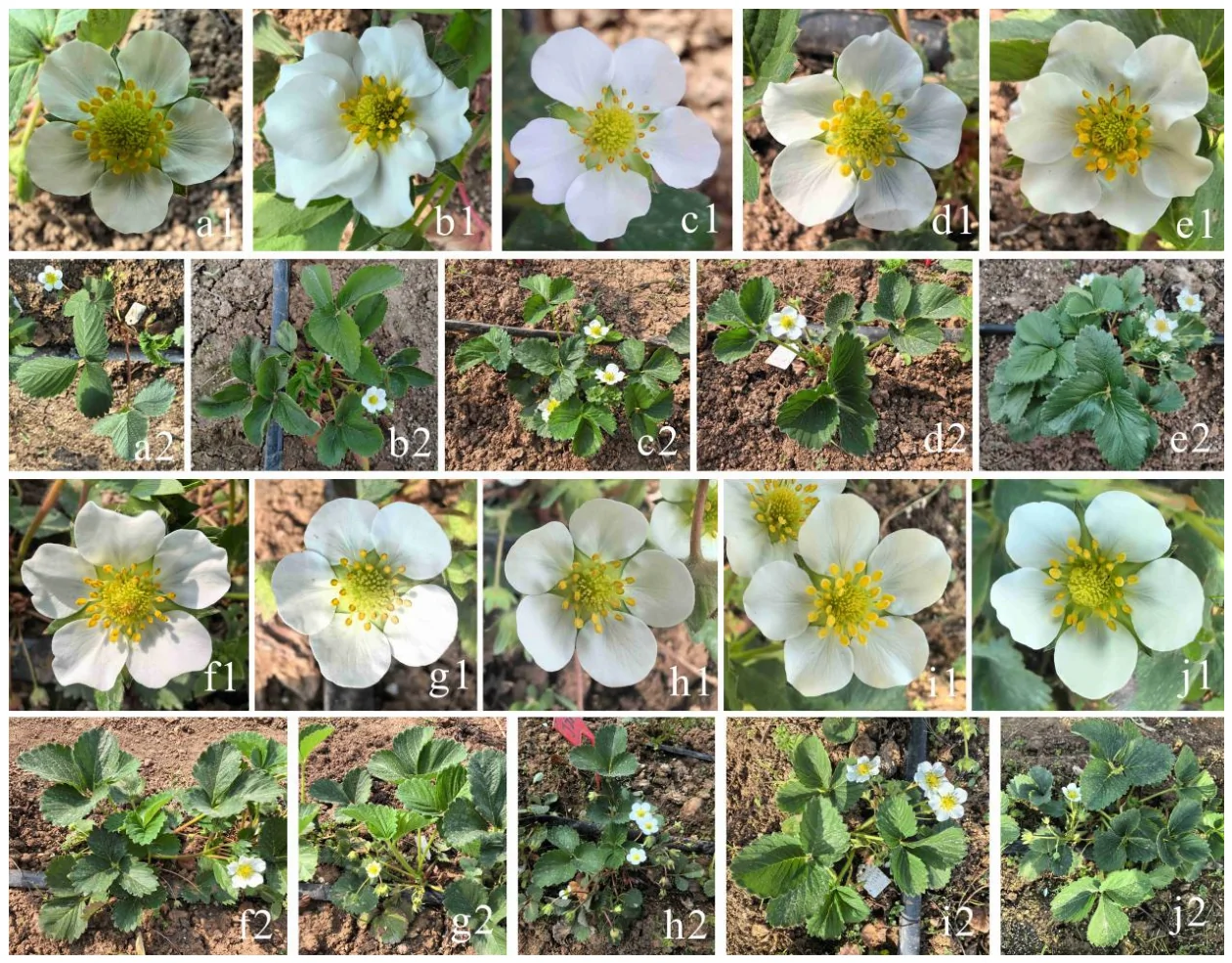
The flowers and plants of some hybrids from ‘Benihoppe’ × ‘Tokun’. (**a1**,**a2**) J2101. (**b1**,**b2**) J2105. (**c1**,**c2**) J2107. (**d1**,**d2**) J2125. (**e1**,**e2**) J2138. (**f1**,**f2**) J2139. (**g1**,**g2**) J2150. (**h1**,**h2**) J2151. (**i1**,**i2**) J2153. (**j1**,**j2**) J2156.

The 27 selected hybrids showed substantial pollen viability, with in vitro pollen germination rates ranging from 21.79% (J2106) to 59.87% (J2136) (Table 4). The flower diameter of J2139 and J2153 was comparable to that of ‘Tokun’ (Figure 5(f1,i1)). All hybrids exhibited higher flower diameter than the lower-value parent ‘Benihoppe’. However, none of the hybrids produced more inflorescences per plant than the female parent ‘Benihoppe’ (Table 5). J2105 displays a double-flowered phenotype. Most hybrids exhibited good stolon propagation ability, whereas three hybrids (J2122, J2127, and J2140) showed relatively poor capacity for stolon production.

**Figure 6:**
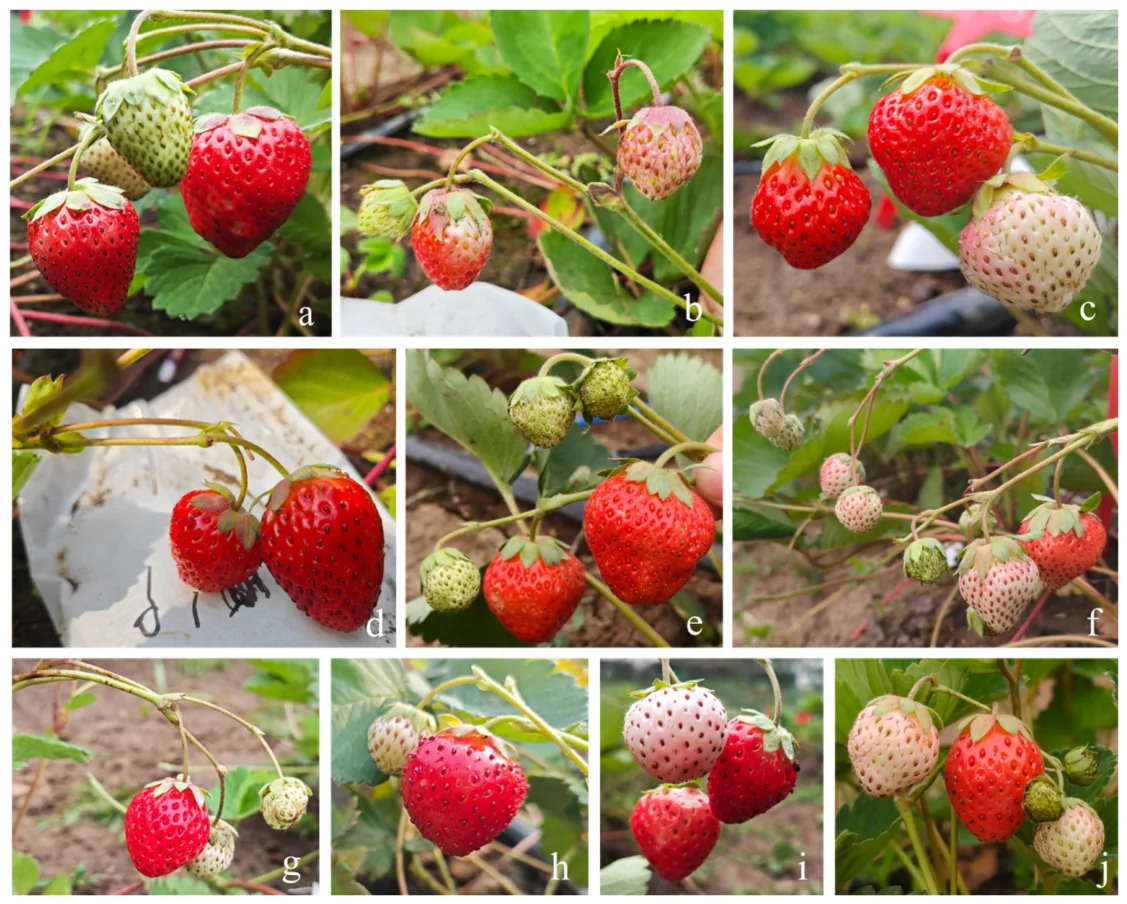
Fruits of some hybrids from ‘Benihoppe’ × ‘Tokun’. (**a**) J2101. (**b**) J2105. (**c**) J2107. (**d**) J2125. (**e**) J2138. (**f**) J2139. (**g**) J2150. (**h**) J2151. (**i**) J2153. (**j**) J2156.

‘Benihoppe’ and ‘Tokun’ exhibited conical and ovoid fruit shapes, respectively. However, their hybrids displayed a variety of fruit shapes, including globular, ovoid, cuneate, short conical, conical, and malformed types. The fruit color of the ‘Benihoppe’ was red, while that of ‘Tokun’ was pink. Most hybrids produced red fruits, though some yielded orange-red (e.g., J2139) or pink fruits (e.g., J2153) (Figure 6). The flesh color of ‘Benihoppe’ was red, whereas ‘Tokun’ had white flesh. Hybrids J2105, J2139 and J2153 also showed white flesh. Most hybrids bore small or abnormally shaped fruits. Nearly all hybrids inherited a distinct peachy aroma from the parent ‘Tokun’. For example, J2138 (2*n* = 9*x *− 2 = 61), J2139 (2*n* = 9*x *− 2 = 61), J2151 (2*n* = 9*x *− 1 = 62), and J2156 (2*n* = 9*x* = 63) exhibited an intense peachy aroma similar to ‘Tokun’. These results indicate that ‘Tokun’ can serve as a valuable parent for developing novel germplasm with this distinctive aromatic trait.

Most hybrids exhibited low values for both average single-fruit weight and plant yield, though a few produced medium-to-large fruits. For instance, J2138 (11.45 g) showed a greater fruit weight than the higher-weight parent ‘Tokun’ (9.60 g), indicating that large-fruited individuals can be effectively selected from this cross. Conversely, some selections such as J2105 yielded exhibited smaller fruits than the parent cultivars. J2101, J2138 and J2153 exhibited plant yields comparable to the parents, whereas the other hybrids showed lower yields than the parents. The flesh firmness of the male parent ‘Tokun’ was only 0.79 kg · (cm)^−2^, significantly lower (*p* < 0.05) than that of the female parent ‘Benihoppe’. All hybrids displayed intermediate flesh firmness between the two parents. Quantitative analysis showed that soluble solids content in most hybrids was close to the mid-parent value, while J2151 and J2156 showed higher than their high-parent values. The majority of hybrids also had higher titratable acidity than both parents. Unfortunately, none of the hybrids surpassed the high-parent lines in vitamin C content.

In summary, five promising lines with aneuploid or nonaploid chromosome constitutions (J2138, J2139, J2151, J2153, and J2156) were obtained through interploidy hybridization, all of which exhibited improved fruit traits. Some of these selected lines displayed more intense aroma, higher flesh firmness, or elevated soluble solids content compared to their parents (Table 5). These results indicate their potential for commercial application and for use in breeding modern aneuploid or nonaploid strawberry cultivars.

## 3. Discussion

The present results showed that ‘Tokun’ is an aneuploid strawberry (2*n* = 10*x* − 3 = 67), which is reported for the first time, differing from previous studies that considered it as a decaploid [15]. Among the 27 hybrids with minimal fruit malformation, the majority were aneuploids and exhibited a peachy aroma. This is likely attributable to abnormal meiosis in the aneuploid ‘Tokun’. It provides a novel insight for aneuploid strawberry breeding.

In the present study, crosses using the octoploid as the maternal parent produced a greater number of fertile offspring. In contrast, the reciprocal cross yielded seeds, but the resulting seedlings died. The discrepancy in crossability between the reciprocal crosses reflects unilateral incompatibility at the gametophytic stage [28,29]. Furthermore, the seedling lethality observed in the latter cross suggests the potential presence of postzygotic isolation. This phenomenon may be preliminarily explained by the Endosperm Balance Number (EBN) hypothesis [30,31]. In distant or interploidy crosses, an imbalance in parental genomic dosage can lead to abnormal endosperm development, consequently compromising seedling survival. However, the underlying cause requires further investigation.

Aneuploid strawberries, characterized by the gain or loss of one to a few chromosomes, exhibit extensive morphological and physiological variation. Although most such changes are harmful [32,33], certain aneuploids may achieve a new genetic balance and display desirable horticultural traits, making them candidates for new cultivars via vegetative propagation [34,35,36,37,38,39,40,41,42,43]. Aneuploidy is thought to enhance evolvability through global physiological and genomic changes [44,45,46], and plants are remarkably tolerant to its effects [35]. This has facilitated the development of ornamental traits such as larger flowers and reduced fertility [47], as well as novel traits like peachy aroma in strawberry hybrids [48]. Thus, aneuploidy induces considerable variation in morphological and agronomic traits, thereby enriching the diversity available for cultivar development. Strawberry is easily propagated by stolons, making it possible to multiply promising aneuploid lines.

Aneuploids can typically be obtained through interspecific hybridization followed by backcrossing [49,50]. For example, Bringhurst and Gill [51] obtained some aneuploids from *F. chiloensis* × *F. vesca* hybrids backcrossed to *F. chiloensis*, with aneuploid gametes derived from the pentaploid, hexaploid, and enneaploid hybrids, and euploid gametes from the octoploid male parent. In the progeny of diploid *F. vesca* × *octoploid F.* × *ananassa*, one individual was aneuploid (2*n* = 8*x* + 2 = 58), which arose from an unreduced 8*x* pollen grain and an incomplete egg [52]. Abnormal gametes were frequently observed in strawberry [52], which may lead to the formation of aneuploids. ‘Tokun’ itself was derived from interspecific hybridization between *F. nilgerrensis* and cultivated strawberry, followed by chromosome doubling and subsequent hybridization events. Given that ‘Benihoppe’ is a regular octoploid that undergoes normal meiosis and rarely produces aneuploid gametes, the aneuploid gametes observed in the progeny are most likely derived from ‘Tokun’.

The successful introgression of the peachy aroma from ‘Tokun’ into the progeny is noteworthy, particularly given the well-documented difficulty of utilizing *F. nilgerrensis* in conventional breeding due to its diploid nature [51,52]. Unlike typical diploid × octoploid crosses that often result in hybrid failure or small, sterile offspring, ‘Tokun’, as an aneuploid genotype, appears to circumvent this ploidy barrier, likely through the production of viable aneuploid gametes, as supported by the observed chromosome constitutions of the progeny. This observation aligns with the notion that aneuploids can achieve a novel genetic balance that supports fertility and trait transmission [35,38]. Moreover, the recovery of multiple progeny lines with both the peachy aroma and improved fruit traits suggests that the aneuploid condition of ‘Tokun’ does not preclude, and may even facilitate, the recombination of desirable traits from wild and cultivated genomes. These results thus provide direct experimental evidence that interploidy hybridization using an aneuploid bridge parent can effectively overcome the long-standing ploidy barrier associated with diploid wild species, offering a practical way for broadening the genetic and aromatic diversity of cultivated strawberry.

## 4. Materials and Methods

### 4.1. Plant Materials

A total of 26 strawberry cultivars were collected from various growers and research stations. Chromosome counting revealed that one cultivar, ‘Tokun’, was an aneuploid (2*n* = 67). To evaluate its breeding potential, reciprocal crosses were conducted between ‘Tokun’ and the octoploid cultivar ‘Benihoppe’ in January 2023. A total of 30 flowers were pollinated for each reciprocal cross direction. The resulting seeds were collected in April, stored at 4 °C for one month, and sown in May 2023. Seedlings were transplanted into planters in a greenhouse in September 2023. A total of 27 progenies with intense fruit aroma and low incidence of fruit malformation were selected and propagated via stolons during 2024. The remaining 89 seedlings were discarded after screening and were not subjected to further ploidy or chromosome analyses. The 27 selected progeny lines were evaluated for ploidy level and agronomic traits in 2025.

### 4.2. Flow Cytometric Determination of Ploidy Levels

Young strawberry leaves were placed in a Petri dish, and 1000 µL nuclei extraction buffer was added. The leaves were rapidly chopped with a sharp razor blade in a single stroke to form a tissue homogenate, ensuring the leaf tissue remained completely submerged in the extraction buffer throughout the process. The prepared homogenate was filtered through a micro-pore filter, and the filtrate was collected into a 1.5 mL centrifuge tube. The centrifuge tube was wrapped in aluminum foil to protect it from light and kept at 4 °C for 5 min. Subsequently, 500 μL solution was transferred into a sample tube, and 30 µL PI staining solution was added. The sample was stained in the dark on ice for 30 min. After staining, the samples were analyzed using a NovoCyte 2040R flow cytometer (ACEA Biosciences, San Diego, CA, USA).

### 4.3. Chromosome Count

Chromosome preparation was according to Zhou et al. [53] with minor modifications. Young root tips were collected and pretreated with 0.71 mM cycloheximide at room temperature for 2 h. The root tips were then excised and fixed in ethanol/acidic acid (3:1) at 4 °C for at least 12 h. For chromosome preparation, the fixed root tips were washed with distilled water and softened in an enzyme mixture containing 1% (*w*/*v*) cellulase RS (Yakult, Tokyo, Japan) and 1% (*w*/*v*) pectolyase Y-23 (Yakult, Tokyo, Japan) at 37 °C for about 20 min, followed by treatment with 20 μL ultrapure water for 30 min. The meristematic tissue was then transferred to a glass slide, mixed with 20 μL 45% acetic acid, and spread using an anatomical needle on a heating plate at 65 °C until nearly all acetic acid had evaporated.

The slides were washed three times in 2 × SSC (0.3 M NaCl and 0.03 M sodium citrate) for 5 min. They were then incubated in 0.01 M HCl for 2 min, after which 200 μL pepsin stock solution was applied. The slides were covered with coverslips and placed in a humid chamber at 37 °C for 10 min, followed by one wash in 2 × SSC for 5 min. A dehydration series was carried out using 70%, 90%, and 100% ethanol (3 min each), after which the slides were air-dried. After counterstaining with DAPI, chromosomes in somatic cells at metaphase were observed and photographed under a 40 × objective using a fluorescence microscope (ZEISS Scope. A1, Carl Zeiss Microscopy GmbH, Oberkochen, Germany). For each strain, at least three clear metaphase cells were analyzed.

Sampling intensity differed among materials: For ‘Tokun’, one root tip was sampled from each of five plants (five roots in total), and a total of 20 well-spread metaphase cells (across the five root tips) were examined; for each hybrid and for each of the 25 additional strawberry cultivars, three root tips from different runner plants were examined, with at least 5–7 clear metaphase spreads scored per root tip.

### 4.4. Investigation of Pollen Germination and Fruit Characteristics

Fresh pollen grains were placed on a medium consisting of 100 g/L sucrose, 4 g/L agar, 0.1 g/L H_3_BO_3_, and pH 5.8. After incubation at 28 °C for 2–3 h, pollen germination was observed under microscope (ZEISS Scope. A1). The fruit shape, color, and weight of all hybrids and their parents were measured following the method of Zhao [54]. The vitamin C (VC) content was determined according to Luo et al. [17]. Titratable acid (TA) was measured by acid-base titration. Soluble solids (SS) were determined directly in the field using a portable refractometer. Fruit firmness (FF) was measured using a GY-2 pointer-type fruit hardness tester (portable maturity tester) with a 3.5-mm-diameter probe inserted to a depth of 10 mm. Five fruits were measured per biological replicate, with three technical replicate measurements per fruit. The values of these traits are presented as mean ± SE.

The peachy aroma was assessed by sensory evaluation. Ten horticultural researchers scored the aroma intensity of ‘Tokun’, ‘Benihoppe’, and their hybrids on a 0–5 scale: 0 = no aroma, 1 = faint aroma, 2 = moderately weak aroma, 3 = aromatic, 4 = strongly aromatic, and 5 = very strongly aromatic. For example, ‘Tokun’ received a mean score of 5 (very strongly aromatic). The scores were statistically analyzed, and mean values were used to assign aroma grades recorded as –, +, ++, +++, ++++, and +++++.

## 5. Conclusions

In conclusion, this study reports, for the first time, that ‘Tokun’ is an aneuploid (2*n* = 10*x *− 3 = 67) rather than a decaploid and demonstrates its value as a bridge parent in strawberry breeding. ‘Tokun’ produces viable gametes with variable chromosome numbers, enabling fertile interploidy crosses with octoploid cultivars and generating progeny with diverse chromosome constitutions and improved fruit traits, particularly the characteristic peachy aroma. This work offers a practical framework for using aneuploid germplasm to overcome ploidy barriers and broaden flavor-related genetic variation in strawberry breeding.

## Figures and Tables

**Table 1:** The ploidy levels of the 26 strawberry cultivars.

Code	Cultivar	Ploidy Level	Code	Cultivar	Ploidy Level
1	Benihoppe	2*n* = 8*x* = 56	14	Qianmei 1	2*n* = 8*x* = 56
2	Kaorino	2*n* = 8*x* = 56	15	Ninglu	2*n* = 8*x* = 56
3	Honeoye	2*n* = 8*x* = 56	16	Zhongmeixiangyu	2*n* = 8*x* = 56
4	Akihime	2*n* = 8*x* = 56	17	Jiuxiang	2*n* = 8*x* = 56
5	Tochiotome	2*n* = 8*x* = 56	18	Lianmeng 2	2*n* = 8*x* = 56
6	Hatsukoi no Kaori	2*n* = 8*x* = 56	19	Sijiguanjun	2*n* = 8*x* = 56
7	Echigohime	2*n* = 8*x* = 56	20	Ganlu	2*n* = 8*x* = 56
8	Angel Eight	2*n* = 8*x* = 56	21	Huangxiang	2*n* = 8*x* = 56
9	Awayuki	2*n* = 8*x* = 56	22	Yutu	2*n* = 8*x* = 56
10	Chandler	2*n* = 8*x* = 56	23	Guangdian	2*n* = 8*x* = 56
11	Toyonoka	2*n* = 8*x* = 56	24	Yingshu	2*n* = 8*x* = 56
12	Darselect	2*n* = 8*x* = 56	25	Meixiangsha	2*n* = 8*x* = 56
13	Allstar	2*n* = 8*x* = 56	26	Tokun	2*n* = 10*x* −3 = 67

**Table 2:** The fruit-setting rate and germination rate of ‘Benihoppe’ and ‘Tokun’.

Crosses	Flowers (No.)	Fruits (No.)	Rate of Fruit-Setting/%	Seeds (No.)	Achenes per Receptacle (No.)	Seedlings (No.)	Rate of Germination/%
‘Benihoppe’ × ‘Tokun’	30	17	56.67	437	25.71	116	26.54
‘Tokun’ × ‘Benihoppe’	30	8	26.67	157	19.63	15	9.55

**Table 3:** Chromosome numbers and parental contributions in hybrids from ‘Benihoppe’ × ‘Tokun’.

Seedling Code	No. of Chromosomes	Chromosome Contribution by the Female	Chromosome Contribution by the Male
J2107, J2112, J2114, J2122, J2125, J2126, J2132, J2137, J2138, J2139, J2153, J2154, J2155	61	28	33
J2101, J2106, J2110, J2113, J2121, J2127, J2136, J2140, J2142, J2146, J2151	62	28	34
J2105, J2150, J2156	63	28	35
Average	61.63	28.00	33.63

**Table 4:** The pollen viability of the 27 hybrids.

Code	Pollen Viability	Code	Pollen Viability
J2101	31.57 ± 1.68 ghi	J2136	59.87 ± 2.75 a
J2105	28.76 ± 1.96 hij	J2137	45.13 ± 1.97 cd
J2106	21.79 ± 1.49 j	J2138	55.38 ± 2.90 ab
J2107	33.54 ± 3.61 fgh	J2139	40.09 ± 3.51 def
J2110	38.62 ± 3.73 defg	J2140	50.65 ± 2.48 bc
J2112	23.15 ± 1.11 j	J2142	54.96 ± 3.99 ab
J2113	37.15 ± 2.43 efg	J2146	48.80 ± 2.32 bc
J2114	31.65 ± 0.33 ghi	J2150	35.00 ± 0.61 fgh
J2121	37.43 ± 1.07 efg	J2151	36.22 ± 1.72 efgh
J2122	38.96 ± 3.18 defg	J2153	33.62 ± 2.52 fgh
J2125	28.84 ± 2.71 hij	J2154	34.76 ± 1.29 fgh
J2126	29.06 ± 1.86 hij	J2155	35.81 ± 1.12 fgh
J2127	25.48 ± 1.35 ij	J2156	43.92 ± 3.7 cde
J2132	24.89 ± 1.53 ij	--	--

Note: Values with different letters in the same column are significantly different at *p* < 0.05.

**Table 5:** Fruit characteristics of the eight excellent strawberry hybrids and their parents.

Code	Plant Height/cm	Plant Diameter/cm	NFI	Diameter of Flowers/cm	Aroma ^i^	FW/g	FF/ (kg · (cm)^−2^)	SS/%	TA/%	VC/ (mg · (100 g)^−1^)
‘Benihoppe’	18.07 ± 1.39 ab	26.80 ± 3.73 ab	7.33 ± 0.33 a	1.97 ± 0.06 e	+++	8.70 ± 1.32 abc	2.89 ± 0.13 a	11.00 ± 0.26 b	0.39 ± 0.12 d	57.64 ± 1.59 a
‘Tokun’	19.44 ± 0.99 a	27.90 ± 1.60 a	5.33 ± 0.34 b	2.83 ± 0.07 b	+++++	9.60 ± 1.28 ab	0.79 ± 0.25 c	8.50 ± 0.45 d	0.82 ± 0.22 c	41.39 ± 0.99 b
J2101	16.54 ± 0.71 bc	26.34 ± 0.63 abc	3.38 ± 0.32 cd	2.53 ± 0.17 cd	+++++	8.87 ± 1.41 ab	2.30 ± 0.15 ab	9.75 ± 0.88 bcd	0.93 ± 0.12 c	40.22 ± 4.10 b
J2105	15.80 ± 0.99 c	23.15 ± 0.97 cd	2.88 ± 0.29 cd	2.37 ± 0.06 d	++++	4.20 ± 0.83 d	1.99 ± 0.16 b	8.75 ± 0.60 cd	0.87 ± 0.06 c	43.95 ± 3.35 b
J2138	16.33 ± 1.42 bc	19.13 ± 1.37 e	3.67 ± 0.33 c	3.13 ± 0.08 a	++++	11.45 ± 3.38 a	2.40 ± 0.20 ab	9.33 ± 0.60 bcd	0.81 ± 0.15 c	28.86 ± 2.16 cd
J2139	19.26 ± 1.97 a	28.06 ± 2.18 a	3.00 ± 0.32 cd	2.83 ± 0.03 b	++++	8.48 ± 1.11 abc	2.45 ± 0.55 ab	10.50 ± 0.58 bc	1.17 ± 0.00 b	21.29 ± 2.51 d
J2150	19.07 ± 0.77 a	23.97 ± 0.98 bcd	3.67 ± 0.30 c	2.67 ± 0.05 bc	++++	4.86 ± 0.41 cd	2.27 ± 0.10 ab	9.89 ± 0.36 bcd	1.02 ± 0.06 bc	29.13 ± 2.24 cd
J2151	18.08 ± 1.12 ab	22.40 ± 1.05 d	2.50 ± 0.17 d	2.50 ± 0.08 cd	++++	8.59 ± 2.52 abc	1.90 ± 0.19 b	12.83 ± 0.83 a	0.93 ± 0.13 c	31.58 ± 6.52 c
J2153	19.40 ± 1.52 a	24.03 ± 2.47 bcd	5.33 ± 0.67 b	2.83 ± 0.03 b	++++	9.97 ± 1.57 ab	2.53 ± 0.11 ab	8.53 ± 0.54 d	0.82 ± 0.11 c	27.50 ± 5.07 cd
J2156	20.15 ± 0.76 a	29.18 ± 2.94 a	2.83 ± 0.31 cd	2.67 ± 0.06 bc	+++++	7.29 ± 1.34 bcd	2.35 ± 0.30 ab	11.13 ± 0.66 b	1.43 ± 0.22 a	54.43 ± 1.03 a

NFI: number of flowers per inflorescence; ^i^ Aroma was treated as a numerical variable by assigning scores based on intensity: 3 pt = +++, 4 pt = ++++, and 5 pt = +++++; FW: fruit weight; FF: fruit firmness; SS: soluble solids; VC: vitamin C. Values are presented as mean ± standard error. Different lowercase letters within the same column indicate significant differences at *p* < 0.05 according to Duncan’s multiple range test.

## Data Availability

Data will be made available on request.
